# Mesenchymal Stem Cells—Potential Applications in Kidney Diseases

**DOI:** 10.3390/ijms20102462

**Published:** 2019-05-18

**Authors:** Benjamin Bochon, Magdalena Kozubska, Grzegorz Surygała, Agnieszka Witkowska, Roman Kuźniewicz, Władysław Grzeszczak, Grzegorz Wystrychowski

**Affiliations:** 1 Psychiatric Services of Thurgovia, Academic Teaching Hospital of the Medical University of Salzburg, 8596 Münsterlingen, Switzerland; benjaminbochon@yahoo.com; 2 General Practice, 43-426 Dębowiec, Poland; magdalena.kozubska@interia.eu; 3 Regional Blood Donation and Blood Treatment Centre, 40-074 Katowice, Poland; gregorian08@wp.pl; 4 DaVita Dialysis, 42-700 Lubliniec, Poland; witkowskaaga@op.pl; 5 Department of Internal Medicine, Diabetology and Nephrology, School of Medicine with the Division of Dentistry in Zabrze, Medical University of Silesia in Katowice, 41-800 Zabrze, Poland; rkuzniewicz@sum.edu.pl (R.K.); wgrzeszczak@sum.edu.pl (W.G.)

**Keywords:** mesenchymal stem cell, mesodermal stem cell, renal ischemia-reperfusion, inflammation, kidney transplantation, microRNA, extracellular vesicles, exosomes

## Abstract

Mesenchymal stem cells constitute a pool of cells present throughout the lifetime in numerous niches, characteristic of unlimited replication potential and the ability to differentiate into mature cells of mesodermal tissues in vitro. The therapeutic potential of these cells is, however, primarily associated with their capabilities of inhibiting inflammation and initiating tissue regeneration. Owing to these properties, mesenchymal stem cells (derived from the bone marrow, subcutaneous adipose tissue, and increasingly urine) are the subject of research in the settings of kidney diseases in which inflammation plays the key role. The most advanced studies, with the first clinical trials, apply to ischemic acute kidney injury, renal transplantation, lupus and diabetic nephropathies, in which beneficial clinical effects of cells themselves, as well as their culture medium, were observed. The study findings imply that mesenchymal stem cells act predominantly through secreted factors, including, above all, microRNAs contained within extracellular vesicles. Research over the coming years will focus on this secretome as a possible therapeutic agent void of the potential carcinogenicity of the cells.

## 1. Introduction

Chronic kidney disease (CKD) affects ~10% of the general population, leading to the deterioration of the quality of life and premature death due to cardiovascular complications. On one hand, chronic renal insufficiency arises as a consequence of continuous insidious kidney damage and scarring in such common diseases as high blood pressure, diabetes, or nephrolithiasis, and, fortunately not as frequent, various forms of chronic glomerulonephritis. On the other, CKD gets instigated or aggravated with incidents of acute kidney injury (AKI), due to such insults as ischemia, infection, autoimmune reaction or toxins like radiological contrast or drugs. The possibilities of pharmacological prevention or attenuation of chronic renal failure are limited to controlling cardiovascular risk factors (usually not optimal), avoidance of potential renal toxins (often unfeasible), or causal treatment of AKI whenever possible (with variable efficiency and frequent complications). In the light of medical advances in other areas, this shortage of therapeutic options raises understandable frustration among patients and their physicians. Stem cell-based therapies may lead to the expected breakthrough in the treatment of kidney diseases.

## 2. Mesenchymal Stem Cells

### 2.1. Types of Stem Cells

Stem cells, owing to their unique ability to replicate and differentiate into specialized organ cells, provide the tissues with the ability to regenerate and survive most injuries [1]. Four types of these cells are defined according to their differentiation potential. In the embryonic period, the early stages of ontogenesis occur owing to the unlimited abilities of totipotent zygotic cells, replaced over time by pluripotent embryonic cells that differentiate into cells of all three germ layers, but no longer have the ability to differentiate into placental cells [2]. Beyond this phase, pluripotent cells that resemble the embryonic stem cells can be obtained by dedifferentiation of fibroblasts or epithelial cells in vitro (induced pluripotent stem cells) [3]. Throughout the lifetime, somatic stem cells are preserved within numerous niches—multipotent ones, which transform into all cells of a given tissue (e.g., bone marrow progenitor cells) or unipotent ones that can only differentiate into one type of mature cell (e.g., cells of the basal layer of the epidermis) [4]. 

At present, the cells under the most extensive investigations in experimental biology and medicine are mesenchymal (mesodermal) stem cells (MSC) that occur in the human body in mesodermal tissues, including placenta, amniotic fluid, umbilical cord tissues, bone marrow, adipose tissue, testis or lungs [5]. 

### 2.2. Regenerative Properties of MSC

MSC exhibit multipotent properties in vitro—when treated with appropriate chemical compounds they have the ability to differentiate into all mesodermal lineage cells, such as fibroblasts, osteocytes, chondrocytes, adipocytes or myocytes [6]. Few studies indicate that they can also transform into cells of endodermal or ectodermal origin [7]. Whereas this vast differentiation potential is of interest and conceivable use in the ex vivo generation of injured tissue replacements, there is only scarce evidence that MSC takes advantage of it in vivo [8]. Most data show that they rather promote tissue repair processes by the means of cell-to-cell interactions or secreted biomaterial, including antioxidant, antiapoptotic and growth factors (GF), such as Epithelial GF, Vascular Endothelial GF (VEGF), Transforming GF (TGF) α and β, Fibroblast GF, Insulin-like GF type 1, and others [9] that stimulate divisions of local progenitor cells. Studies show that these compounds are released from MSC in the free state or contained within spherical vesicles of a 30–100 nm diameter—exosomes of an endosomal origin or microvesicles budding from the cellular membrane. These extracellular vesicles allow signal transmission between the cells not only through the transported proteins, but also mRNAs and microRNAs [10]. Multiple microRNA particles have been identified within MSC extracellular vesicles and their patterns differ considerably between experimental models of different ischemic/inflammatory diseases [11]. Of great importance is homing of MSC to the ischemic, necrotic or inflamed sites, as a result of their membrane expression of chemokine receptors and integrins [5], responsiveness to damage associated molecular patterns [12] or mitochondria released from dead cells with their engulfment [13]. This tropism reduces the distance of secreted products to their target locations and allows an additional way of restoring local homeostasis by MSC—intercellular transfer of mitochondria [14]. Substituting defective native mitochondria with MSC-derived fully operational ones provides ATP for most needed anabolic reactions [15] and has been shown to revive damaged alveolar or corneal epithelia [16,17]. According to most reports mitochondria are moved to the damaged cells by means of nanotube tunneling [18], microvesicles [19] or cellular fusion, as recently comprehensively reviewed [20]. 

MSC are increasingly used in reconstructive surgery. In countries with less stringent legal restrictions (such as Japan and South Korea), they are used as a replicative matrix for renewal of joint surfaces and regeneration of facial defects (which requires collagen scaffolding) or as a source of cytokines and GFs stimulating natural healing in periodontal disease and skin wounds [21]. Their usefulness is examined in experimental models of corneal damage, lung, spinal cord and brain injuries [22].

### 2.3. Immunomodulatory Properties of MSC

Beside their regenerative potential, MSC are characterized by the ability to modulate immune responses. Of note, they are characterized by low expression of MHC class I antigens and no expression of MHC class II antigens or B7-1, B7-2 and CD40 costimulatory molecules. This implies that the infusion of allogeneic stem cells does not induce a clinically significant immune response [23]. Most importantly, exposure to MSC in vitro or their systemic administration in large amounts (~10^6^–10^8^ cells) inhibits Th17 lymphocytes, augments the pool and activity of regulatory T-cells, and increases expression of anti-inflammatory cytokines like IL-10, subsequently blunting inflammatory reaction [24]. Their use in the treatment of autoimmune diseases, such as inflammatory diseases of the joints [25] or intestines [26] has been tested with good results. Contrary to initial assumptions, studies with infusions of exogenous MSC showed that their anti-inflammatory effect is not primarily the result of the direct interaction with immune cells in the target inflamed tissue, but they can act from distance by the means of their secretome, at least partially contained within exosomes or microvesicles [27]. Meticulously isolated extracellular vesicles of umbilical cord MSC (by means of size-exclusion chromatography) have been shown to exert a potent immunosuppressant effect in vitro, in contrast to other fractions of the MSC conditioned medium [28]. 

A British group has recently found that immunosuppressive activity of human bone marrow MSC in the experimental model of an established severe inflammatory reaction (MSC intravenous infusion on the third day of graft-versus-host disease in mice) relies on their apoptosis. The reductions of the lung and spleen pools of graft-versus-host disease effector T-cells were detectable either when MSC were lysed and engulfed by recipient’s NK or CD8+ T cells in an antigen-independent way or when apoptosis was induced in MSC prior to their infusion. Based on the results with the additional use of an inhibitor of indoleamine 2,3-dioxygenase, the authors concluded that increased expression and release of this anti-inflammatory cytokine by recipient’s phagocytes upon MSC apoptosis is the mediator of the immunosuppressive effect [29]. However, it can be reasoned that this very mechanism does not exclude the role of extracellular vesicles and microRNAs which can be extensively released from the apoptotic cells. Somewhat in line with the latter study, the key role of apoptosis of MSC with the self-activation of IL-1/IL-1R/NFκB pathway induced by caspases, has been implicated in the increased secretion of Prostaglandin E2 by MSC and consequential proinflammatory M1→ anti-inflammatory M2 macrophage transition [30].

### 2.4. Source of MSC for Research Purposes

Mesenchymal stem cells can be obtained from fetal tissues, umbilical cord blood or Wharton’s Jelly, which for obvious reasons limits this route of acquisition to the perinatal period. In males, they may be acquired throughout a lifetime from testis [31], but due to greater accessibility, MSC for research purposes are derived mostly from bone marrow or subcutaneous adipose tissue. Fat may become preferential as a source of MSC not only owing to less invasive procurement, but also due to higher MSC concentration than in bone marrow, lesser expression of MHC class I antigens, and greater replicative and secretory potential of MSC [32,33] (Table 1). MSC can also be obtained from induced pluripotent stem cells by their differentiation in vitro [34], which constitutes another life-long, yet technically much more challenging way of acquisition.

### 2.5. Kidney as a Source of MSC

A promising method of a non-invasive collection of MSC is their isolation from urine. In 2008, for the first time, Zhang et al. from North Carolina identified cells present in the urine in the amount of 2–7/100 mL that adhere to plastic and form colonies of differentiated daughter cells expressing membrane markers characteristic of urothelial, endothelial, and interstitial cells, or myocytes [35]. In further studies, the differentiation of these cells in appropriate culture media to the endo, ecto- and mesodermal lineage was achieved [36]. In contrast to MSC, urine-derived cells (up to 75% of them) show telomerase activity, which is associated with their higher replicative potential, apparently not associated with an increased risk of tumorigenesis [37]. The origin of these cells is most likely glomerular—MSC-like cells with a vast differentiation potential were isolated from the renal cortical decapsulated glomeruli [38] and shown nephroprotective in the renal ischemia-reperfusion injury (IRI) [39]. These cells seem distinct from the renal perivascular MSC-like cells that possess lesser differentiating capabilities (no adipogenesis), but also compelling kidney reparative properties confirmed in the tubular epithelial cell line injury in vitro or non-ischemic AKI in mice [40].

## 3. Research on the Use of Mesenchymal Stem Cells in Kidney Diseases

Potential applications of MSC in kidney diseases primarily take advantage of their secretory capabilities and aim to enhance the natural regenerative processes in the settings of AKI, and in the bolder perspective, even induce such processes in CKD. On the other hand, MSC can be used to grow renal cells in vitro to replace damaged native cells. In this context, cultures of kidney fragments (organoids) with subsequent implantations are to be considered, despite all technical complexity. Thirdly, the use of immunomodulatory properties of MSC can play an important role in the treatment of inflammatory kidney disease, such as primary and secondary glomerulonephritis, or in the prevention of rejection of the transplanted kidney. Finally, urinary isolation of cells that are functionally similar to MSC can significantly increase the availability of the material for all these therapeutic options.

### 3.1. Attempts to Replace Damaged Kidney Tissue

Kidney organogenesis includes mutually stimulating processes of the growth and differentiation of the intermediate mesoderm cells—to the ureteric bud (mesonephric duct protrusion) and to the metanephric blastema. They further transform respectively into the urinary tract system (up to connecting tubules), or nephrons, renal interstitium and endothelium [41]. Development of a new kidney by recapitulating organogenesis in whole or in part in vitro is an intensively studied area of tissue engineering. As research from the 1990s showed, appropriate sets of GFs can induce early stages of the development of an isolated ureteric bud, as well as metanephric blastema [42,43]. However, it remains problematic to derive cells with fetal characteristics from an adult, as well as to provide vascularization of the developing tissue. The first issue is currently being investigated with the use of induced pluripotent cells obtained from fibroblasts. In several cases, these cells (as well as embryonic MSC) were successfully cultured over <4 weeks into three-dimensional organoids with structural and functional characteristics of nephron complexes. The applied protocols included the use of such stimulants as Fibroblast GF-9, WNT-signaling pathway agonist, and activin [44,45]. Recent works by van den Berg et al. showed that renal organoids generated from human embryonic or induced pluripotent stem cells, became efficiently vascularized upon kidney subcapsular implantation in mice. Compared to organoids cultured continuously in vitro, those that were placed in vivo on the 18th day of the three-dimensional growth featured a more advanced structural maturation regarding glomerular endothelium, filtration barrier (slit diaphragm formation), tubular epithelium polarization and differentiation, as well as peritubular vascularization, when assessed on the 28th day since implantation [46]. 

A different way of generating a kidney replacement is through colonization of an acellular connective tissue scaffold with cells of a high replicative and differentiating potential or mature renal cells. The use of mature renal cells would eliminate the possible risk of cancer associated with stem cell divisions, but is much more technically difficult. A more feasible approach is through intra-arterial and intra-ureteral infusions of multipotent cells, which in response to the scaffold environment and native or exogenous GFs would differentiate into glomerular endothelial and epithelial or tubular cells, respectively. In order to obtain an intact acellular, sterile connective tissue scaffold of the kidney, an organ retrieved from another organism is infused with detergents like sodium lauryl sulfate or nonionic surfactants [47]. A successful repopulation of digested rat kidneys with rat fetal cells has been reported by the authors from Boston in 2013. The umbilical vein endothelial cells infused into renal artery produced endothelial layer in the entire renal circulation, whereas neonatal kidney cell suspension administered into the ureter resulted in the settlement of cells in their physiological niches of the urinary tract, beginning from podocytes down to connecting tubules. Such regenerated kidneys perfused in vitro with a solution containing crystalloids, glucose, albumin, amino acids, creatinine and urea showed partial functionality in the production of "urine", creatinine filtration (10–25% of the physiological level) and albumin retention (47% of the physiological level). In contrast, after orthotopic implantation, despite adequate blood flow and absence of bleeding or clotting and urine production of ~1/3 of physiological volume, they featured only a negligible excretion of urea and creatinine [48]. Similar results were obtained by another team of researchers from China [49]. On the other hand, Italian researchers did not manage to obtain sealed layers of cells in the distal parts of the renal circulation and proximal sections of the nephrons despite various protocols of embryonic MSC administration [50]. This area of research is awaiting verification of the usefulness of bone marrow or adipose MSC.

In the context of kidney regeneration, it is of note, that Iranian authors intend to assess effects of intravenous infusion of autologous bone marrow MSC on the course of autosomal dominant polycystic kidney disease [51].

### 3.2. Induction of Repair Processes after Acute Kidney Injury

One of the major study areas of MSC has been their influence on the course of renal IRI, being the most frequent cause of AKI and occurring in the clinical settings of shock, cardiac arrest, extracorporeal circulation and peritransplantation period. In addition to apoptosis caused by an energy deficiency and acidosis during ischemia, reperfusion results in further tissue damage, due to oxidative stress and inflammatory reaction. Studies conducted so far have shown that MSC infusion alleviates IRI of the kidney. Regardless of the mode of MSC administration (to the renal artery or intravenously, at various times in relation to IRI), the animal models showed a milder course of acute kidney failure [52], with reductions of oxidative damage and local expression of inflammatory cytokines [53], increased renal pool of regulatory T lymphocytes [54], faster regeneration of renal tubular epithelium [55], and reduction of subsequent fibrosis of the renal interstitium [56]. 

Intravenous infusion of MSC (derived from induced pluripotent stem cells) was equally nephroprotective in the model of toxin-induced AKI. In mice 2 × 10^5^ MSC, injected 2 h after administration of Adriamycin, mitigated proteinuria and renal failure present on day 7 in controls. This could be attributed to the observed inhibition of oxidative stress and apoptosis in the tubular cells [57]. 

As already mentioned, these beneficial effects of MSC result from their secretory properties, not replicative-differentiating potential. In an experiment conducted by a German-American team, rats subjected to 40-min ischemia of both kidneys were administered labelled allogenic bone marrow-derived MSC (10^6^ cells) to the aorta immediately after or 24 h after renal reperfusion. In both cases, two hours after the end of the infusion, MSC were found in the renal tissue (within the glomerular and peritubular capillaries), but were not detected, neither did differentiate to other cells, during the subsequent 22 and 70 h of observation. Nevertheless, faster normalization of renal excretory function, reduced renal expression of proinflammatory cytokines (Interleukin-1β, Tumor Necrosis Factor α, Interferon γ) and higher renal expression of anti-inflammatory and antiapoptotic factors, such as Interleukin-10, basic Fibroblast GF, TGF α and Bcl-2 were observed at the conclusion of observation [58]. 

The fraction of MSC secretome responsible for this nephroprotective effect may be largely RNA, as shown by Italian researchers. Microvesicles isolated from human bone marrow MSC medium (30 μg), administered intravenously to rats after a 45-min ischemia of the sole kidney, attenuated acute renal failure and atrophy of tubular cells, whereas subjecting these microvesicles to RNase abolished their beneficial effects in this experimental model [59]. Further studies in rodents and cell lines by this largely Torino-based group revealed that the nephroprotective effect of microvesicles derived from bone marrow MSC in AKI may be owing to high contents of a few microRNA families (miR-483–5p, -191, -28–3p, -423–5p, -744, -129–3p, -24, and miR-148a) that get transferred to tubular epithelial cells. This results in altered expression of at least 165 genes involved in cellular adhesion and extracellular matrix remodeling, including downregulation of the transcription of fibrinogen-α subunit [60]. Furthermore, extracellular vesicles secreted by the bone marrow MSC were shown by this group to be heterogeneous in size and contents, with the exosomal fraction to diminish apoptosis and enhance proliferation of tubular cells undergoing hypoxia/reperfusion in vitro. This fraction was rich in the microRNA families involved in kidney regeneration (miR-100, -21, -24, -214, -34a, -127, -30c, -29a, -125b, -10b, -let-7c, -99a, -17 and miR-20a) [61]. Another study showed that also the above mentioned glomerular MSC-like cells (obtained from human renal cortex) and their extracellular vesicles alleviate AKI in mice by promoting tubular cell proliferation when infused intravenously at reperfusion following a 35-min sole kidney ischemia (10^5^ cells or 400 × 10^6^ vesicles, respectively). Interestingly, the injected vesicles were homing to the injured tubular cells (and not glomeruli) where they were visible for up to 6 h after infusion, contrary to the administered vesicles obtained from dermal fibroblasts, which did not accumulate in the kidney and consequently showed no effects whatsoever. In line with earlier studies, the nephroprotective properties of the vesicles derived from the glomerular MSC-like cells were eliminated in case they had been pretreated with high-concentration RNase and 62 microRNAs were found to be specifically abundant in these extracellular vesicles [39].

Similar to MSC, the MSC-derived extracellular vesicles were found to protect kidneys also from a toxic injury. The above quoted Italian group of Camussi showed that human bone marrow MSC (75 × 10^3^) or their microvesicles (15 μg) alleviated AKI to the same extent when administered intravenously on the third day after glycerol injection in mice. Moreover MSC microvesicles were homing and getting incorporated into tubular cells in vivo only in glycerol-exposed animals, and not in controls, and their antiapoptotic effects were RNA-dependent [62]. Correspondingly positive renal outcomes were obtained by these authors in the mouse model of cisplatin-induced lethal AKI, in which MSC-secreted microvesicles (100 μg) were infused 8 h after cisplatin injection. Importantly, nearly half of the animals were alive after three weeks, and when injections of the microvesicles were repeated every four days, 80% of mice survived [63]. 

The very same model of toxic AKI was used by German investigators to show that the renoprotective properties of MSC secretome can be enhanced by hypoxic preconditioning of the MSC culture. Such treatment of mouse adipose MSC (0.5% oxygen for 48 h) increased their expression of VEGF and by >2-fold its secretion, as well as that of other 63 proteins. This corresponded with a moderately alleviated course of AKI following infusion of the hypoxia-preconditioned MSC medium (at 24 h after cisplatin injection), as compared to the non-manipulated MSC medium [64]. Significantly positive renal outcomes were obtained by another group in the rat model of renal IRI with the administration of hypoxia-preconditioned (1% oxygen for 24 h) human adipose MSC at reperfusion. Like in the former study, these cells showed higher expression of VEGF than naïve MSC. Furthermore, in vivo they featured greater antioxidant and antiapoptotic properties [65]. 

More light on the possible cellular mechanisms of the regenerative properties of the MSC extracellular vesicles was shed by Chinese groups. In studies conducted in the rat models of renal IRI, urologists from Shanghai have shown that 100 µg microvesicles derived from the human umbilical cord mesenchyme alleviated kidney macrophage infiltration, tubular apoptosis and AKI when intravenously infused at reperfusion. This may be due to the revealed attenuation of the renal expression of fractalkine (itself a potent chemoattractant), likely mediated by a transfer of certain microRNAs to renal cells [66]. RNA transfer from MSC extracellular vesicles to tubular cells was also shown by these authors to underlie the increase of tubular VEGF synthesis and attenuation of AKI, as well as renal fibrosis, in rats that were administered the vesicles at reperfusion of the solitary kidney [67]. Other experiments showed that additional effectors of the MSC vesicles in the tubular cell nuclei of the kidneys subjected to IRI may be Nrf2/antioxidant response element with subsequent overexpression of antioxidant enzymes [68] or the Sox9 transcription factor enhancing tubular cell proliferation and diminishing kidney fibrosis [69] (one of the few studies with the use of human adipose-derived MSC and their vesicles).

On the other hand, another group from the same university have convincingly shown that a transfer of protein may also take part in the nephroprotective effects of MSC and their vesicles. It was found by these researchers that extracellular vesicles derived from human induced pluripotent stem cell-derived MSC exert a potent nephroprotective effect in the renal IRI by a transfer of protein that inhibits programmed inflammatory cell death (necroptosis) [70]. 10^12^ extracellular vesicles delivered intravenously 1 h before bilateral 30-min kidney ischemia decreased the kidney histological damage and the degree of renal failure in rats at 48 h after ischemia. Analyses in vitro showed that this phenomenon relied on a transfer of the transcription factor Specificity protein-1 to the renal proximal tubule cells with subsequent activation of nuclear expression of sphingosine kinase-1. This enzyme phosphorylates dihydrosphingosine into sphinganine-1-phosphate, a compound shown to alleviate the extent of IRI [71]. 

The use of MSC in the clinical setting of renal ischemia (not related to kidney transplantation) was the subject of a study conducted by researchers from Minnesota. Fourteen patients with unilateral renal artery stenosis were administered autologous adipose-derived MSC (10^5^ or 2.5 × 10^5^ cells/kg body weight) to the stenotic renal artery. After the subsequent three months blood flows increased in both the stenotic and the contralateral kidney, and glomerular filtration was higher by 21% compared to the control group [72]. Somewhat contrary to these results, MSC were ineffective in the setting of postoperative AKI that occurred within 24 h from cardiac surgery. Intraaortic infusion of allogenic bone marrow-derived MSC (2 × 10^6^ cells / kg body weight within 48 h from the operation) in 67 patients did not improve kidney function nor 30-day mortality. In fact, patients who received the MSC suspension showed a tendency to a worse prognosis in the postoperative period [73]. As noted by the authors, such results indicate that MSC may not be as effective in the environment of an established inflammatory reaction as in its prevention, like with the pretreatment of anticipated ischemic AKI. Nevertheless, a clinical trial is planned by a team from Massachusetts in patients with AKI treated with continuous renal replacement therapy, in which patient’s blood will be exposed to MSC across a semipermeable membrane of a hollow fiber extracorporeal device inserted into the hemodiafiltration circuit [74]. This would prevent any MSC-induced immunization, eliminate the risk of uncontrolled MSC replication, but expectantly provide a constant influx of the cells’ beneficial products into the patient.

### 3.3. Immunomodulation of Kidney Transplantation

IRI is an inherent element of kidney transplantation and is manifested in the peritransplant period as the delayed graft function. The therapeutic potential of MSC in this setting is additionally related to a possible immunosuppressive effect, which may increase the effectiveness of pharmacological prophylaxis of the transplant rejection. The animal studies and scarce observations in humans, despite varying protocols of application, encourage the use of MSC-based therapies in kidney transplant patients, with no clear preference of any of the cell sources (autologous, donor-derived, third-party).

In rats in which allogeneic or syngeneic kidney transplantation was performed, infusion of allogeneic bone marrow MSC into the graft artery during reperfusion reduced the organ infiltration with CD8+ lymphocytes and monocytes, and alleviated failure of the rejected graft [75]. MSC were likewise effective with intravenous administrations. Syngeneic MSC infused in this way during kidney transplantation reduced the expression of inflammatory cytokines in the graft in rats [76]. In mice MSC administered intravenously 24 h before kidney transplantation increased the pool of regulatory T-cells in the spleen and prolonged survival of the transplanted kidney (which was not observed with the infusion performed at 24 h post-transplantation) [77]. In addition, Spanish researchers reported that MSCs can also be effective in the treatment of chronic graft nephropathy—intravenous infusion at 11 weeks after renal transplantation in rats resulted in reduced proteinuria, diminished inflammatory infiltration of the interstitium, and lesser interstitial fibrosis/tubular atrophy at 24 weeks after organ transplantation [78].

In one of the pioneer studies of human MSC use in renal transplantation, adipose MSC derived from the perirenal fat of the living kidney donor or the third-party MSC, inhibited similarly both pre- and post-transplant anti-donor and anti-third party alloreactivity of recipient’s T lymphocytes [79]. This finding was followed by the first clinical studies of the MSC use in the living-donor kidney transplant recipients conducted in Italy. In total, two patients underwent intravenous administration of autologous bone marrow MSC at one week after transplantation (1.7 × 10^6^ and 2.0 × 10^6^ cells per kg body weight, respectively), while the other two were given autologous MSC 24 h prior to kidney graft implantation (2.0 × 10^6^ cells per kg body weight intravenously). Over the five- to seven-year follow-up the mean renal function yearly decline rate was lower by ~70% than in non-MSC treated transplanted patients [80]. However, the MSC recipients showed considerable variability in the clinical course with one patient developing calcineurin inhibitor-free graft tolerance, whilst the other one experiencing acute graft rejection at two weeks after transplantation—both patients being ones that were given MSC before kidney implantation. Nevertheless, there was no elevation in the frequency of infections or neoplasms in the MSC-treated subjects. With the exception of one patient, a ~50% reduction in the blood percentage of memory CD8+ T cells was observed at 12 months post-transplantation compared with the pre-transplant levels, a phenomenon not seen in any of the controls [80].

In another pilot study, authors from China infused donor-derived bone marrow MSC into the graft renal artery during reperfusion and intravenously at one month after kidney transplantation in six recipients. This allowed the reduction of tacrolimus dosing by ~50% (with C0 ~4 vs. ~7 μg/L in controls) without episodes of rejection within 12 months of observation [81]. The immunosuppressive efficacy of MSC in the late post-transplant period was studied by Dutch researchers, who applied autologous bone marrow MSC in six kidney transplant recipients with subclinical rejection or histological progression of graft nephropathy at six months after transplantation. Two intravenous infusions of 10^6^ cells/kg body weight each, in a week interval, eliminated peri-tubular cellular infiltrates at 12 months after transplantation, and patients’ blood monocytes were characterized by diminished replication in vitro. It is notable, however, that CMV or BKV infection occurred in three of these patients [82].

In the largest clinical trial conducted so far 105 Chinese renal transplant recipients were administered autologous MSC at graft reperfusion and again after two weeks in place of anti-IL-2 receptor antibodies. Such induction of immunosuppression was associated with faster organ regeneration in the first month after transplantation, as well as a lower rate of cellular rejection (7.6% vs. 21.6% in the control group) and its milder course in the six-month follow-up [83].

On the other hand, studies appeared that denied effects of intravenous infusions of MSC on the kidney transplant outcome—improvement of renal allograft function and rat survival was found only when allogeneic fat MSC were injected into the graft artery, and not when they were administered intravenously at implantation [84]. Even more discouraging are the recent findings of another Chinese team of researchers, who injected allogeneic umbilical cord blood MSC to 21 recipients intravenously immediately prior to transplantation (2 × 10^6^/kg body weight) and, additionally, to graft artery at reperfusion (5 × 10^6^), on top of the standard immunosuppression. In the period of one-year follow-up, no statistically significant differences were found against the controls in terms of postoperative and infectious complications, renal function, frequency of rejection nor survival time of the kidney transplant [85]. Of note, one experimental study from Germany also found unfavorable effects of MSC infusion in the peritransplant period—rats given syngeneic or donor-derived bone marrow MSC intravenously four days before kidney transplantation showed symptoms of more severe cellular and humoral rejection and worse graft function on the 10th day after graft implantation [86].

### 3.4. Immunomodulation of Primary Glomerulonephritis

Inflammatory glomerulopathies constitute another area of potential clinical applications of the MSC. The few conducted experimental studies have shown their beneficial effect on the course of these diseases. For example, in an animal model of membranoproliferative glomerulonephritis, intravenous infusion of allogeneic fetal MSC reduced glomerular expression of proinflammatory cytokines, decreased monocyte infiltrates, mesangial hyperplasia, synthesis of connective tissue matrix and proteinuria. Interestingly, in this study the MSC culture medium inhibited mesangial expression of TNFα and monocyte chemoattractant protein 1 (MCP-1) in vitro [87]. Likewise, in the rat model of focal segmental glomerulosclerosis (doxorubicin-induced nephropathy), several intravenous infusions of bone marrow MSC increased glomerular VEGF synthesis, which was accompanied by attenuations of: Glomerular monocyte infiltration, apoptosis of the podocytes, and the extent of podocyte-parietal epithelial bridging [88].

A key role of MSC secretome in their actions was revealed in a rat model of experimental anti-glomerular basement membrane disease, in which intraperitoneal administration of human MSC medium over the 10 days since induction of disease reduced renal proinflammatory cytokine expression, increased plasma MCP-1 concentration and shifted the glomerular macrophage infiltration into the dominance of the anti-inflammatory M2 cells. This was associated with the lesser formation of crescents, reduction of proteinuria and improvement of glomerular filtration [89]. Similar favorable results were obtained in rats that received human MSC intravenously on the fourth day of the same type of rapidly progressive glomerulonephritis. Beside a smaller degree of histological and functional renal disorders, increased expression of anti-inflammatory cytokines, as well as reduced TGF β, collagen I and III mRNA concentrations in the kidney cortex were found in these rats on day 13 of the disease [90].

The use of MSC in primary glomerulonephritis in human patients has been described in two case reports from Italy. In the first one, a 13-year-old boy experienced a relapse of focal segmental glomerulosclerosis in the kidney graft on the second day after the transplantation. Intensification of immunosuppressive therapy with rituximab did not bring remission and the patient underwent weekly plasmapheresis, which only temporarily reduced the amount of proteinuria. Therefore, at month 7 after the transplantation, the patient was given two intravenous infusions of allogeneic bone marrow MSC (1 × 10^6^ cells/kg body weight each), which was repeated after further three and seven months. In the 22-month follow-up from the first infusion, proteinuria remained stable without the need to perform plasmapheresis, the plasma concentration of Epidermal GF and TGF α decreased, and serum creatinine oscillated around 0.9 mg/dL [91]. In the second case, autologous bone marrow MSC (1.5 × 10^6^ cells/kg body weight) were given intravenously to a 73-year-old patient with pANCA-positive rapidly progressive glomerulonephritis, whose treatment with steroid and cyclophosphamide was ineffective, and rituximab was discontinued due to severe oral candidiasis. Seven days after administration of MSC, serum creatinine decreased from 7.8 to 2.2 mg/dL, which was accompanied by normalization of urinary sediment, a significant reduction in the pANCA titer, and decrease in serum cytokine concentrations, as well as an increase in the regulatory T-cell pool in the blood. The MSC infusion was repeated after eight months, due to the recurrence of the disease with similar efficiency (serum creatinine 1.9 mg/dL), and over the next 11 months of observation, the patient’s condition was good and did not require any treatment [92].

### 3.5. Immunomodulation of Lupus Nephritis

Systemic lupus erythematosus is a multisystem condition that involves kidneys in approximately 60% of cases. Since the standard immunosuppressive treatment is mostly insufficient in patients with severe proliferative lupus nephritis [93], the cell-based therapies are a promising alternative owing to their immunomodulatory properties. To date, many pre-clinical studies regarding the use of MSC transplantation in the context of lupus nephritis therapy have been performed giving mostly positive outcomes in terms of proteinuria and renal histopathology, as reviewed lately [94]. In one of the most recent experimental studies, Tani et al. applied systemic treatment with low-dose allogenic bone marrow MSC (10^6^ cells/kg body weight intravenously) in a mouse model of spontaneously developing lupus with co-occurring glomerulonephritis. The therapeutic strategy was the early MSC administration (18–22 weeks of age) in order to investigate its potential interference with the developing disease, as well as to compare the outcomes of a single and multiple cell infusions. MSC treatment resulted in a significant delay of proteinuria appearance with the most beneficial results in mice that received multiple cell administrations. Nevertheless, histopathological nephritis scores did not differ from the controls and some harmful effects of MSC were observed, such as significantly higher B-cell deposition in kidneys of mice that received multiple MSC doses and decreased levels of regulatory T-cells after both single and multiple MSC injections [95].

The MSC-based therapies are used increasingly in Chinese patients with lupus nephritis, and often with good outcomes in terms of clinical remission [96] or blood Treg/Th17 balance [97]. However, there is still a shortage of randomized, double-blind, placebo-controlled trials. In 2017 Deng et al. presented a randomized clinical study comparing the efficacy of a standard immunosuppressive treatment (methylprednisolone and cyclophosphamide applied intravenously, followed by maintenance oral prednisolone and mycophenolate mofetil) with (*n* = 12) or without (*n* = 6) a co-administration of human umbilical cord MSC (two intravenous injections of 2 × 10^8^ cells in total) [98]. The primary endpoint was remission of nephritis (combined partial and complete remission) defined with specified values of serum creatinine, urinary red blood cells and proteinuria in the 12-month follow-up. Remission was noted in 75% of patients in the MSC-treated group and in 83% of patients in the placebo group. The reduction of proteinuria was comparable and no significant difference in serum creatinine levels between the two groups was noted. When it comes to secondary endpoints (clinical symptom scores, complement concentration, anti-dsDNA antibody and ANA titers, death and commencement of permanent dialysis or renal transplantation), no significant differences were observed, either, and the trial was terminated ahead of schedule. The newest report regarding the application of MSC in the lupus nephritis came from Spain and suggests the efficacy of the cells in the most severe cases. Three patients who demonstrated class IV active proliferative lupus nephritis, were treated with allogenic bone marrow MCS (9 × 10^7^ of cells infused intravenously) at the exacerbation of the disease [99]. One week after MSC infusion a considerable decrease of proteinuria was observed in all patients and maintained throughout the course of a nine-month follow-up. The complete clinical symptom remission in two patients and partial remission to the mild activity of the disease in the third patient were noted and call for a randomized and controlled trial in such patients.

Of note, so far no animal or clinical studies have been reported with the application of MSC extracellular vesicles in the lupus nephropathy, although the rationale for such investigations have been formulated [100,101].

### 3.6. Therapeutic Potential in Diabetic Kidney Disease

Glomerular microinflammation takes part in the pathogenesis of diabetic nephropathy, albeit is not the target of standard immunosuppressive treatment, due to its small intensity and possible metabolic complications of such therapies. Not surprisingly, the interest of researchers has recently focused on MSC and the studies of their use in diabetic nephropathy are consequently, and somewhat paradoxically, more advanced than in gross inflammatory glomerulopathies.

On one hand, MSC can indirectly prevent kidney damage or inhibit its progression by improving glycemic control of diabetes, as shown in experimental and clinical studies. In the mouse model of established streptozotocin-induced type 1 diabetes, intravenous administrations of human bone marrow MSC or their medium induced regeneration of pancreatic islets and subsequently reduced blood glucose levels by 30–35% [102,103]. MSC may also hinder type 2 diabetes: Myoblasts pre-exposed to the MSC medium featured lower expression of proinflammatory cytokines, increased synthesis and expression of the GLUT4 glucose transporter, and consequently less compromised insulin sensitivity upon 24-h exposure to a palmitate solution. MSC medium was as effective in this regard as a metformin solution [104]. The influence of MSC on the course of type 2 diabetes in humans has been evaluated so far in several studies conducted in small groups of patients, and with considerable methodological differences—in terms of the origin of administered cells, dose and route of administration (intravenous, pancreatic artery), or the use of controls. In the majority of these works, increases in the blood C-peptide concentrations and reductions of hemoglobin A1c levels were observed for several months after the MSC infusions, with no effects on the peripheral insulin resistance [105,106].

The nephroprotective properties of MSC in diabetic nephropathy have been revealed in experimental models of type 1 diabetes. Intravenous infusion of allogeneic bone marrow MSC in the late phase of streptozotocin-induced diabetes resulted in the reduction of albuminuria and the degree of glomerular filtration impairment in rodents. In the renal tissue of these animals, reduced oxidative stress, as well as diminished expressions of proinflammatory cytokines, apoptotic proteins and TGF β were observed, whereas expressions of nephrin, podocin, bone morphogenetic protein 7 and VEGF were augmented [107,108].

The immunomodulatory effects of MSC-secreted factors, rather than the cells themselves, have been implicated by a study in mice with streptozotocin-induced or high-fat diet-induced diabetes. In both models, intravenous infusions of both rat bone marrow MSC or their medium reduced alike renal proinflammatory cytokine expression and macrophage infiltration. This was accompanied by attenuated albuminuria and diminished interstitial fibrosis [109]. The key role of the MSC-secreted extracellular vesicles could be deduced from only scarce renal localization of the administered MSC and the fact of obtaining comparable beneficial histological effects in the kidney after subcapsular administration of exosomes previously isolated from MSC [109]. The nephroprotective effects of factors secreted by the MSC were also indicated by the results of a study in rats with streptozotocin-induced diabetes that were injected intravenously with exosomes derived from the aforementioned pluripotent MSC-like cells isolated from human urine. In these animals, no mesangial expansion, reduced renal expression of apoptotic proteins, as well as diminished albuminuria were found in comparison to the control group [110]. Recently, Ebrahim et al. succeeded in clarifying the mechanisms of the beneficial effects of bone marrow MSC exosomes in type 1 diabetic nephropathy in rats, showing their capability of improving tubular cell autophagy, as seen with the electron microscopy and reflected in the reduced renal expression of the mechanistic target of rapamycin. This was accompanied by significantly reduced expression of fibronectin and TGF β with diminished fibrosis and improved function of the kidneys [111].

In the years 2015–2016, the first reports emerged on the use of allogeneic multipotent mesenchymal precursor cells in patients with type 2 diabetes. The cellular suspension was obtained from the bone marrow by a selection of cells with membrane expression of alkaline phosphatase STRO-3 (rexlemestrocel-L, currently in the second phases of clinical verification in groups of patients with various medical conditions). In the first of these works, these cells were given in the amount of 0.3–2 × 10^6^/kg body weight to 45 patients with inadequately controlled type 2 diabetes. In the second one the same preparation was given to patients with diabetic renal insufficiency (eGFR 20–50 mL/min/1.73 m^2^) at a dose of 150 × 10^6^ or 300 × 10^6^ cells (both groups numbering 10 patients). During the 12 weeks following infusions, no significant side effects or immunization of patients with donor antigens were noted. In this relatively short period of observation, there was however no significant effect of the tested preparation on the clinical parameters related to diabetes and renal failure [112,113].

## 4. Conclusions

The presented review of published works on the use of mesenchymal stem cells in kidney diseases shows the greatest advancement of experimental research in the fields of AKI, kidney transplantation, and diabetic or lupus nephropathies (Table 2). The majority of results indicate the reparative, immunosuppressive and antifibrotic effects of factors released from MSC in the environment of low-grade inflammation (as in the case of diabetic glomerulopathy) or in prevention/alleviation of a developing inflammatory injury (as with pretreatment of the anticipated ischemic AKI, early treatment of ischemic or toxic tubular injury or administration preceding/concurrent with kidney graft implantation). The efficacy of MSC secretome in the milieu of an established renal inflammation or injury (as with post-IRI administration) seems less uniform.

**Figure 1:**
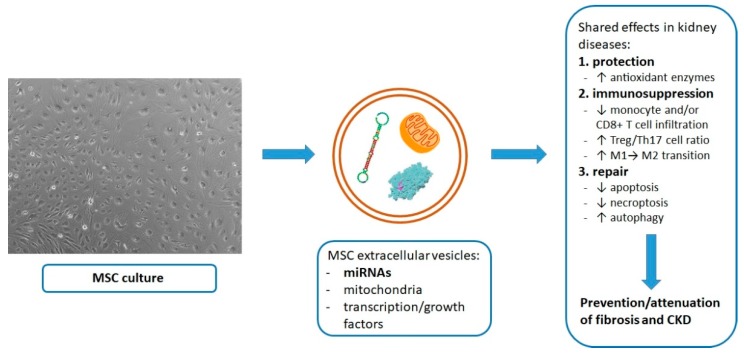
Shared major mechanisms of nephroprotection with exogenous MSC/MSC-derived products in renal diseases.

The most promising MSC product in the context of renal regeneration/immunosuppression appears to be microRNAs contained within extracellular vesicles (Figure 1). Membranous protection enables their homing to the injured tissue and subsequent epigenetic modulation of the local expression of reparative cytokines and transcription/growth factors. Studies show that also MSC-secreted proteins or mitochondria take part in tissue regeneration. However, the role of freely released transcription/growth factors or cell-to-cell mitochondrial transfer may be limited to MSC infused to the aorta or renal artery, for the assured proximity to the injured cells. On the other hand, the intravenously administered MSC, which largely get trapped and apoptotic in the lungs, may dispatch both proteins and mitochondria within extracellular vesicles that shall be able to reach the injured or inflamed kidneys. Of importance for the future clinical applications, the secretory reparative potential of MSC can be enhanced in culture, as with hypoxic preconditioning.

It is striking that the number of clinical trials with the use of MSC in kidney diseases so far has remained disproportionately low considering their therapeutic potential emerging from experimental studies (placebo-controlled trials are particularly in demand). For example, in the last three years there has been only one reported use of MSC in renal transplant recipients [85]. This may be related to the fact that the outcomes of the pioneer uses of MSC in patients have not been uniformly favorable. Also, and likely more importantly, shortage of trials can be attributed to reservations that both scientists and bioethical boards may have towards applications of allogenic cells with high mitotic potential, as such associated with the risk of immunization or cancer.

Thus far, there have been findings of MSC enhancing divisions in cancer cell lines [114] and augmenting the metastatic potential of co-administered cancerous cells in animals [115]. Nevertheless, there has been no report of de novo carcinogenesis in vivo following MSC infusion, neither in animals nor in humans. One reported case of angiomyeloproliferative renal lesions was related to percutaneous renal injections of not MSC, but peripheral blood-derived autologous hematopoietic cells [116]. Moreover, it has been shown that MSC can actually inhibit the progression of cancerous tumors. In hamsters with induced premalignant stages of squamous cell carcinoma of the mouth cavity, they decreased the progression of lesions (except for the largest doses) [117]. Less optimistic are the latest discoveries in the field of MSC immunogenicity. Contrary to the assumptions of its negligibility due to lack of expression of HLA class II antigens, equine bone marrow stromal cells treated with a proinflammatory cytokine (Interferon γ) expressed MHC class II antigens on their cellular membrane and stimulated the proliferation of T lymphocytes in vitro [118]. It is not certain whether this process also takes place in vivo—current applications in humans do not indicate significant immunogenicity of allogeneic MSC, although it is necessary to bear in mind the relatively short periods of observation in the conducted studies [119].

All these objections direct the researchers’ interest into microvesicles or exosomes secreted by MSC. In particular, microRNAs contained within are considered equally efficient, but potentially not tumorigenic and less immunogenic therapeutic objects. Although increasingly used in animal models, their applicability in clinical trials is dulled by the lack of sufficient knowledge of the consequences of administering exogenous molecules with such high stability as microRNAs [120]. On the other hand, it has to be noted that this very characteristic may constitute the observed effectiveness of systemically administered MSC-derived vesicles in kidney disease models. 

Regardless of all question marks, the secretory products of mesenchymal stem cells deserve further research in experimental and subsequently clinical studies, providing a chance for the most awaited breakthrough in the treatment of the inflammatory and, especially, ever more frequent autoimmune diseases with renal involvement. Finally, commenting on the concerns of possible side effects of MSC-based therapies, it can be questioned whether the widely used classical immunosuppressive drugs—source of common infectious, metabolic and cancerous complications—would ever be approved for the clinical use at the present-day level of safety expectations.

## Figures and Tables

**Table 1:** Differences in the properties of bone marrow and fat mesenchymal stem cells.

Differentiating Characteristic	Bone Marrow MSC	Adipose MSC
Stability in culture	Lower	Higher
Aging	More advanced	Less advanced
Replicative potential	Lower	Higher
Immunomodulatory properties	Lower	Higher

**Table 2:** Intensity and outcomes of the studies of MSC or MSC secretome in the major renal settings.

Kidney Disease Setting		Animal Studies		Human Studies	
		MSC	MSC Medium or EVs	MSC	MSC Medium or EVs
Acute kidney injury	ischemic	↑↑↑	↑↑↑	↑↓	○
	non-ischemic	↑↑	↑↑	○	○
Kidney transplantation	pre-/intra-implantation	↑↑	○	↑↑	○
	post-implantation	↓	○	↑	○
Chronic allograft nephropathy		↑	○	↑	○
Glomerulo-nephritis	primary	↑↑	↑	↑	○
	lupus	↑↑↑	○	↑↓	○
Diabetic kidney disease		↑↑	↑↑	○	○

MSC—mesenchymal stem cells; EVs—extracellular vesicles; ○ no conducted studies; ↑ single conducted study or a few case reports, positive outcomes; ↑↑ several conducted studies, mostly positive outcomes; ↑↑↑ numerous conducted studies, mostly positive outcomes; ↑↓ several conducted studies, conflicting outcomes; ↓ single conducted study, negative outcomes.

## Abbreviations

ANA	Anti-Nuclear Antibody
AKI	Acute Kidney Injury
CKD	Chronic Kidney Disease
dsDNA	double stranded Deoxyribonucleic Acid
eGFR	estimated Glomerular Filtration Rate
HLA	Human Leukocyte Antigen
IRI	Ischemia-Reperfusion Injury
MCP	Monocyte Chemoattractant Protein
MHC	Major Histocompatibility Complex
MSC	Mesenchymal (mesodermal) stem cells
pANCA	perinuclear Anti-Neutrophil Cytoplasmic Antibody
RNA	Ribonucleic Acid
TGF	Transforming Growth Factor
VEGF	Vascular Endothelial Growth Factor
